# Non-invasive quantification of myocardial fibrosis in diabetic mice using in-vivo high-resolution MRI

**DOI:** 10.1186/1532-429X-11-S1-P69

**Published:** 2009-01-28

**Authors:** Sok Sithikun, Frank Kober, Alexis Jacquier, Jérôme Kalifa, Francis Kopp, Marie-France Bonzi, Jean-Claude Deharo, Patrick J Cozzone, Monique Bernard

**Affiliations:** 1 CRMBM, Marseille, France; 2 Laboratory of Histology, Marseille, France; 3 Department of Cardiology, Arrhythmias unit, Marseille, France

**Keywords:** Diabetic Mouse, Myocardial Fibrosis, Diabetic Cardiomyopathy, Relaxation Time Measurement, Vertical Scanner

## Introduction

There is an established role for magnetic resonance imaging in the assessment of myocardial fibrosis, in ischaemic and non-ischaemic cardiomyopathies. The amount of fibrosis is of importance because of its prognostic value, in terms of arrhythmias occurrence, and haemodynamic consequences.

T2 relaxation time directly depends on physico-chemical properties of each tissue. Thus, myocardial T2 time determination can help in quantifying fibrosis. Diabetic cardiomyopathy is characterized by myocardial structural modifications including hypertrophy, microcirculation impairment and interstitial fibrosis.

## Purpose

To describe a non-invasive MRI method using high-resolution myocardial T2 time measurement to assess myocardial fibrosis in a model of diabetic mice in vivo.

## Methods

Two multi-slice spin-echo sequences were performed for T2 time assessment respectively at 20 and 9 ms echo time (resolution 85 × 85 μm2, slice thickness 1.0 mm, imaging time 15 minutes), in ten 16-week old C57Bl/6J after 8 weeks of streptozotocin-induced diabetes, and ten control mice, under isoflurane anesthesia using a 11.75 T vertical scanner (Bruker, Rheinstetten, Germany). All images were acquired in short-axis view. MRI measurements were then compared with histological quantification of fibrosis using picrosirius red staining.

## Results

T2 relaxation time was significantly lower in diabetic mice (13.8 +/- 2.8 ms versus 18.9 +/- 2.3 ms in the control group, p < 0.05). This was associated with a significant increase of myocardial fibrosis as evaluated by picro-sirius red staining, in diabetic mice.

## Conclusion

We describe here a non-invasive and accurate MRI method to quantify myocardial fibrosis in vivo in diabetic mice, using T2 relaxation time measurement. This method can be applied in many ischaemic or non-ischaemic cardiomyopathies in which a fibrotic phenomenon is involved.

